# Endocrine paraneoplastic syndromes in patients with neuroendocrine neoplasms

**DOI:** 10.1007/s12020-018-1773-3

**Published:** 2018-10-02

**Authors:** Kosmas Daskalakis, Eleftherios Chatzelis, Marina Tsoli, Nektaria Papadopoulou-Marketou, Georgios K. Dimitriadis, Apostolos V. Tsolakis, Gregory Kaltsas

**Affiliations:** 1 Department of Surgical Sciences, Uppsala University, Uppsala, Sweden; 2 1st Department of Propaupedic Internal Medicine, Endocrine Oncology Unit, Laiko Hospital, National and Kapodistrian University of Athens, Athens, Greece; 3 251 Hellenic Air Force and VA General Hospital, Athens, Greece; 4 Division of Endocrinology Department of Medical and Health Sciences, Linkoping University, Linkoping, Sweden; 5 Warwickshire Institute for the Study of Diabetes Endocrinology and Metabolism (WISDEM) Arden NET CoE and Human Metabolism Research Unit (HMRU), University Hospitals of Coventry and Warwickshire, NHS Trust, Coventry, CV2 2DX UK; 6 Department of Oncology and Pathology, Karolinska Institute, Stockholm, Sweden; 7 Cancer Center Karolinska, CCK, Karolinska University Hospital Solna, R8:04, Stockholm, Sweden; 8 Department of Medical Sciences, Uppsala University, Uppsala, Sweden

**Keywords:** Paraneoplastic syndrome, Neuroendocrine tumours, Ectopic Cushing’s syndrome, Hypercalcitonaemia, PTHrP secretion

## Abstract

**Objective:**

Our aim was to assess the prevalence of endocrine paraneoplastic syndromes (EPNS) in neuroendocrine neoplasms (NENs) and estimate its impact on patient outcomes.

**Design:**

This is a retrospective analysis of 834 patients with NENs (611 gastrointestinal, 166 thoracic, 57 of unknown and various other primary origin). We included 719 consecutive NEN patients treated at EKPA-Laiko Hospital, Athens, Greece and 115 patients with lung carcinoid (LC) treated at Uppsala University Hospital, Uppsala, Sweden. EPNS diagnosis was based on standard criteria.

**Methods:**

Twenty-one patients with EPNS were detected: 16 with ectopic Cushing’s syndrome (ECS), one with hypercalcaemia due to parathyroid hormone-related protein (PTHrP) secretion, three with hypercalcitonaemia and one patient with dual secretion of calcitonin and beta-human chorionic gonadotropin (β-HCG). All tumours were well-differentiated; 10 patients had Stage IV disease at diagnosis.

**Results:**

The prevalence of EPNS in the Greek cohort was 1.9%, whereas that of ECS among LC patients in both centres was 6.7%. Median overall survival (OS) for patients with EPNS was 160.7 months (95%CI, 86–235.4) and median event-free survival (EFS) was 25.9 months (95%CI, 0–57.2). Patients presenting with EPNS prior to NEN diagnosis had longer EFS compared to patients with synchronous or metachronous EPNS (log-rank *P* = 0.013). Patients with ECS of extra-thoracic origin demonstrated shorter OS and EFS compared to patients with ECS of lung or thymic origin (log-rank *P* = 0.001 and *P* < 0.001, respectively). LC patients with and without ECS were comparable in 5-year and 10-year OS rates (66.7% and 33.3% versus 89.8% and 60.2%, respectively; 95%CI [189.6–300.4 months], log-rank *P* = 0.94) and in median EFS, 67 versus 183 months, 95%CI [50.5–207.5], log-rank *P* = 0.12).

**Conclusion:**

EPNS are relatively rare in patients with NENs and mainly concern well-differentiated tumours of the foregut. Among patients with EPNS, LC-related ECS may not adversely affect patient outcomes when diagnosed prior to NEN and effectively been treated.

## Introduction

Paraneoplasmatic syndromes (PNS) can be produced by peptides, amines or cytokines secreted by the tumour, or by immune cross-reactivity between neoplastic and normal tissues, and originate from either endocrine or non-endocrine neoplasms [1]. Neoplasms that cause endocrine PNS (EPNS) exhibit a wide range of malignant potential, ranging from benign to highly malignant tumours. The majority of EPNS is produced from non-endocrine neoplasms and are found in tumours with an adverse biological behaviour, involving the lungs, breast, prostate, ovaries, skin, as well as certain haematological neoplasias [2–6]. Generally, in highly malignant neoplasms, disease prognosis is determined from the biological behaviour of the underlying malignancy rather than the related EPNS manifestations.

Neuroendocrine neoplasms (NENs) have also been associated with EPNS [7]. These neoplasms comprise a heterogeneous group of mostly well-differentiated tumours with a generally favourable prognosis. They can often be “functioning”, i.e. they share the ability to synthesise and secrete bioactive substances characteristic of the cell of origin that may cause distinct clinical syndromes [8]. In contrast, EPNS constitute a distinct array of manifestations that cannot be attributed to a secreting neoplastic lesion related to the originating anatomical site. Thus, the secretion of bioactive substances is considered ectopic. A large number of different EPNS secondary to NENs has been reported, exhibiting clinical signs and symptoms that are similar to those caused when the secretory product is derived from the expected site of origin [9].

Importantly, EPNS might complicate the patient’s clinical course and response to therapy, and may potentially impact prognosis. Occasionally, EPNS manifest themselves prior to the diagnosis of the underlying neoplasia [10]. However, the development of EPNS does not always correlate with tumour stage, grade, and patient outcomes [11]. Thus, the prognostic impact of EPNS in NENs remains to be elucidated further, particularly as there is an increment in the number of NENs diagnosed compared to other neoplasias.

To date, systemic documentation of NEN-related EPNS although is increasingly been recorded still remains rather limited and relevant studies are widely dispersed [9, 12–15]. The aim of this study was to record EPNS secondary to NENs and to provide information regarding EPNS prevalence, clinical presentation and impact on overall prognosis in patients with NENs. As previous studies have suggested that EPNS are more prevalent in lung compared to other NENs, we opted to address this particular issue by including a large number of such neoplasms.

## Subjects and methods

Data were extracted retrospectively from the clinical files and electronic medical records of NEN patients admitted at EKPA-Laiko Hospital, Athens, Greece, as well as from patients with LC treated at Uppsala University Hospital, Uppsala, Sweden, using a commonly agreed protocol. Only patients with definite histopathological classification as NENs and a diagnosis of EPNS were included; patients with small or large cell lung carcinomas were excluded. Specifically, the medical records from 719 consecutive patients with various NENs treated at EKPA-Laiko Hospital, and 115 patients with LC treated at Uppsala University Hospital were reviewed for the presence of EPNS (Supplementary Table 1).

The diagnoses were made between 1994 and 2017 and patients were followed until death or the 1 May 2018. Demographics [age, gender] as well as disease characteristics (type and timing of EPNS in relation to NEN diagnosis, NEN type, stage and grade, immunohistochemical marker status, serum biomarkers status, octreoscan/(68)Ga-DOTATATE positron emission tomography (PET) and ^18^F-fluorodeoxyglucose (FDG)-PET positivity) were registered. NEN progression was assessed radiologically according to the Response Evaluation Criteria in Solid Tumours (RECIST 1) [16]. In order to assess the impact of EPNS on overall prognosis in patients with NENs, event-free survival (EFS) was calculated from the NEN diagnosis for patients exhibiting disease recurrence or progression. For the subset of patients receiving several lines of treatments, we assessed recurrence or progression in relation to the first line of treatment, which mainly consisted of resective surgery. The presence of EPNS was recorded either prior to or at NEN diagnosis (synchronous) or subsequently during the course of the follow-up (metachronous). For ECS diagnosed prior to the identification of the ectopic tumoural source of adrenocorticotropic hormone (ACTH), the syndrome is defined as overt when it is followed by a prompt identification of the tumoural source and covert when the tumour is identified during a subsequent evaluation or a prolonged follow-up [17].

The study was conducted according the 1975 Declaration of Helsinki and approved by the regional ethics review boards in Athens, Greece, and Uppsala, Sweden. Written informed consent was obtained from all the study participants. To ensure the quality of data reporting, we followed the STROBE statement [18].

### Diagnosis of NEN and EPNS

Diagnosis of NEN was made on the basis of histopathological confirmation, cross-sectional and functional imaging. The role of the serum biomarker Chromogranin-A in NEN diagnosis was supplementary. EPNS diagnosis was made according to standard criteria as presented in Supplementary Table 3 [19]. Specifically, the parathyroid hormone-related protein (PTHrP) hypersecretion diagnosis was based on persistent hypercalcaemia and suppressed plasma PTH levels in combination with elevated plasma PTHrP levels. Cushing’s syndrome secondary to ectopic ACTH secretion (ectopic Cushing’s syndrome (ECS)] was defined as ACTH-dependent hypercortisolaemia in patients with tumours known to be associated with ectopic ACTH secretion, and was substantiated with further endocrine tests that suggested an ectopic source and/or positive ACTH immunostaining of non-pituitary tumours. Hypercalcitonaemia EPNS diagnosis was based on plasma elevation of calcitonin >10 pg/ml in patients with tumours known to be associated with ectopic calcitonin secretion, exclusion of other conditions related to high calcitonin levels (including normal thyroid ultrasound), presence of symptoms not attributed to the secretion of another measurable bioactive compound (flushing and/or diarrhoea) and positive calcitonin immunostaining of non-thyroid NENs.

Carcinoid syndrome is rare in patients with pancreatic NEN (panNEN), but serotonin secretion may occur, as demonstrated by elevated urinary 5-hydroxyindoleacetic acid (U-5-HIAA) levels [20]. However, in this setting serotonin secretion is considered as eutopic; thus patients with panNENs and elevated 5-HIAA levels were not included in this study [20].

Patients regularly underwent cross-sectional imaging with computed tomography (CT) or magnetic resonance tomography (MRT), and functional imaging with somatostatin receptor scintigraphy and/or PET using different tracers, both at baseline and during follow-up, in order to assess tumour load and to evaluate treatment effects and disease recurrence or progression as indicated per patient.

Patients with NENs were screened at diagnosis for eutopic hormone secretion according to the site of origin, i.e. C-peptide, gastrin, glucagon, insulin, pancreatic polypeptide, pro-insulin, VIP, 24-h U-5-HIAA and chromogranin A for panNENs.

U-5-HIAA and chromogranin A were also evaluated in patients with LCs and small intestinal NENs. Patients with other NENs as well as NENs of unknown primary from the Greek database (EKPA-Laiko) underwent extensive hormonal screening, including beta human chorionic gonadotropin (β-HCG). All patients with panNENs, LCs, thymic carcinoids and UPO NENs were screened for calcitonin at diagnosis. However, ectopic hormonal screening was not performed on a regular basis during follow-up, but as indicated per patient on the basis of relevant clinical and biochemical findings. ACTH, plasma and 24-h urinary cortisol were analysed when Cushingoid symptoms/signs were present. PTHrP was evaluated only in cases with hypercalcaemia, whereas other hormones were evaluated in the presence of suggestive symptoms. When different assays of the same hormone were employed over the time period of the study, values were converted to relative units.

We used the 2017 and 2015 WHO classification systems for grading of gastroenteropancreatic NENs and thoracic NENs (thymic and lung carcinoids), respectively [21, 22]. Accordingly, for staging, the 8th edition of the American Joint Committee on Cancer (AJCC) and the revised 8th edition of Thoracic Neoplasms Stage Classification were used [23, 24].

As NENs of thoracic origin (lung and thymic) are related to frequencies as high as 45% of ECS prevalence secondary to NENs [15], we performed a separate analysis regarding primary and secondary end-points in this particular group.

### Statistics

All statistical analyses (frequencies, descriptive statistics, Kaplan–Meier curves, log-rank tests] were done with the SPSS 23.0 software package [IBM SPSS Statistics, Armonk, NY, USA). Overall and EFS were analysed using Kaplan–Meier methods. Log-rank testing (Mantel–Cox) was used to determine whether there was a difference between mortality and disease recurrence or progression in NEN patients with EPNS, stratified by EPNS type and timing, origin of the primary tumour, as well as within the subset of LC patients with ECS compared with the Swedish cohort of LC patients, with complete follow-up data. Tests were two-sided and *P*-value < 0.05 was considered statistically significant.

## Results

From a total of 834 NEN patients, 21 patients (9 women, 42.9%) met the diagnostic criteria of EPNS (Supplementary Table 2). Sixteen patients presented with ECS (76.2% of EPNS), one patient had hypercalcaemia due to PTHrP secretion, three patients had hypercalcitonaemia in the absence of thyroid disease, and one male patient demonstrated dual ectopic secretion of calcitonin and β-HCG. The prevalence of EPNS in the Greek cohort of NENs was 1.9% (14/719), whereas the prevalence of ECS among patients with LCs in both centres was 6.7% (11/164). ECS was the main EPNS detected in our series (Table 1).

**Table 1:** Patient characteristics at diagnosis

*Sex ratio* (M:F)	8:5
*Median age at diagnosis (years)*	56 (range: 25-75)
*E* *PNS type*	
Ectopic Cushing’s syndrome	16
Hypercalcitonemia	4
Hypercalcemia due to PTHrP secretion	1
Ectopic β-HCG secretion	1
*Site of primary*	
Small intestine	1
Pancreas	5
Lung (histopathological type)	13 (7 typical/ 6 atypical)
Thymic	1
Unknown	2
*Syndromic:sporadic ratio*	0:21
*TNM stage at diagnosis*	
I	6
II	3
III	3
IV	9
*WHO grade*	
G1	5
G2	15
G3	1
*Timing of ePNS in relation to NEN diagnosis*	
Prior (overt:covert ratio)	10 (6:4)
Synchronous	6
Metachronous	5
*Immunohistochemical positivity*	
ACTH	14
Calcitonin	4
PTHrP	1
β-HCG	1
*Serum biomarker elevation*	
ACTH	16
Calcitonin	4
PTHrP	1
β-HCG	1
*Octreoscan/68Gallium-PET* (positive:negative ratio)	12:9
*FDG-PET* (positive:negative:not performed ratio)	5:3:13

All tumours were well differentiated (5G1, 15G2 and 1G3) and none occurred in a familial setting. Nine patients presented with Stage IV disease, including all four patients with hypercalcitonaemia and the patient with hypercalcaemia due to PTHrP secretion. Within the subset of LC patients (*n* = 12), seven tumours had atypical histopathology; one patient had hypercalcitoneamia, whereas 11 patients manifested ECS. ECS was of thoracic origin in 12 patients (11 lung/1 thymic) and extra-thoracic origin in four patients (2 pancreatic NENs (panNENs)/1 small intestinal NEN/1 NEN of unknown primary origin). In those patients with LC-related ECS, one presented with stage IV disease, whereas the remaining 10 patients as well as the one patient with thymic-related ECS presented at stages I–III. PanNENs were involved in three different EPNS with a prevalence of 1.9% (5/265) and were mainly disseminated at diagnosis (4/5). The different NEN sources of EPNS are presented in Table 1.

The median age at NEN diagnosis was 56 years (range: 25–75) whereas the diagnosis of EPNS was synchronous in six patients, metachronous in five patients and prior to the NEN diagnosis in the remaining 10 patients, all of whom presented with ECS (six overt and four covert ECS cases, Table 1, Supplementary Figure 1).

**Fig. 1:**
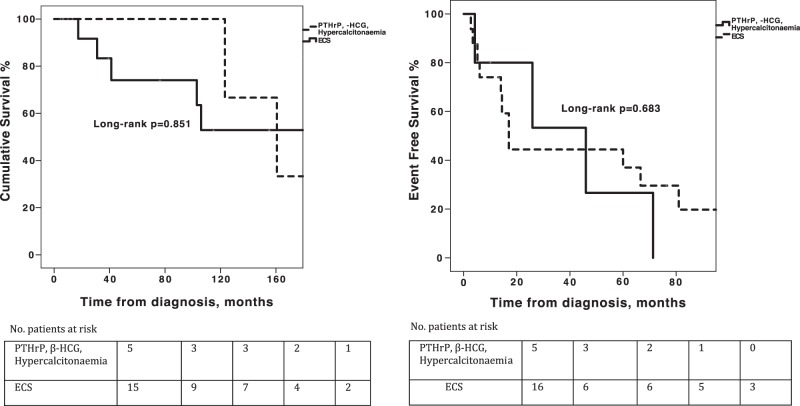
**a** Overall survival and **b** event-free survival analysis of patients with paraneoplastic endocrine syndromes (EPNS) secondary to neuroendocrine neoplasms stratified by type of EPNS

**Fig. 2:**
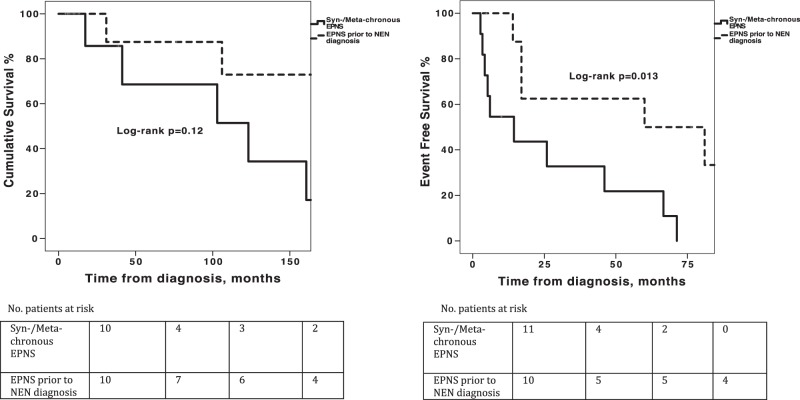
**a** Overall survival and **b** event-free survival analysis of patients with paraneoplastic endocrine syndromes secondary to neuroendocrine neoplasms stratified by timing of diagnosis

**Fig. 3:**
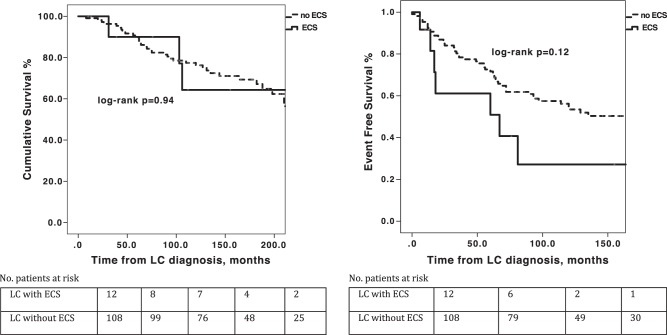
**a** Overall survival and **b** event-free survival analysis of lung carcinoid patients with and without ectopic Cushing’s syndrome

Median follow-up was 89.6 months [range 5–236 months]. Median overall survival (OS) for NEN patients with EPNS was 160.7 months (95%CI, 86–235.4) and median EFS was 25.9 months (95%CI, 0–57.2). Patients with different types of EPNS (ECS versus other EPNS types) were comparable in OS and EFS (log-rank *P* = 0.851 and 0.683, respectively, Fig. 1). Patients presenting with EPNS prior to NEN diagnosis had longer EFS compared to patients with synchronous or metachronous EPNS (log-rank *P* = 0.013, Fig. 2). Within the subset of LCs, patients with and without ECS were comparable in 5-year and 10-year OS rates (66.7% and 33.3% versus 89.8% and 60.2%, respectively; 95%CI [189.6–300.4 months], log-rank *P* = 0.94), as well as in the median (EFS 67 versus 183 months; 95%CI [50.5–207.5], log-rank *P* = 0.12, Fig. 3). However, patients with ECS of extra-thoracic origin demonstrated shorter OS and EFS compared to patients with ECS of lung or thymic origin (log-rank *P* = 0.001 and *P* < 0.001, respectively).

All patients underwent cross-sectional imaging at diagnosis of NEN disease with nine patients having Stage IV disease at baseline, whereas six patients were diagnosed at Stage I, three patients at Stage II and three patients at Stage III. Only one patient with EPNS prior to the NEN diagnosis had Stage IV disease at baseline. Functional imaging undertaken at baseline and/or during the NEN disease course demonstrated that 12 patients had uptake on octreotide scintigraphy or 68 Gallium PET imaging, whereas nine patients were negative. A subset of eight patients underwent FDG-PET imaging, exhibiting tracer uptake in five cases (Table 1).

**Fig. 4:**
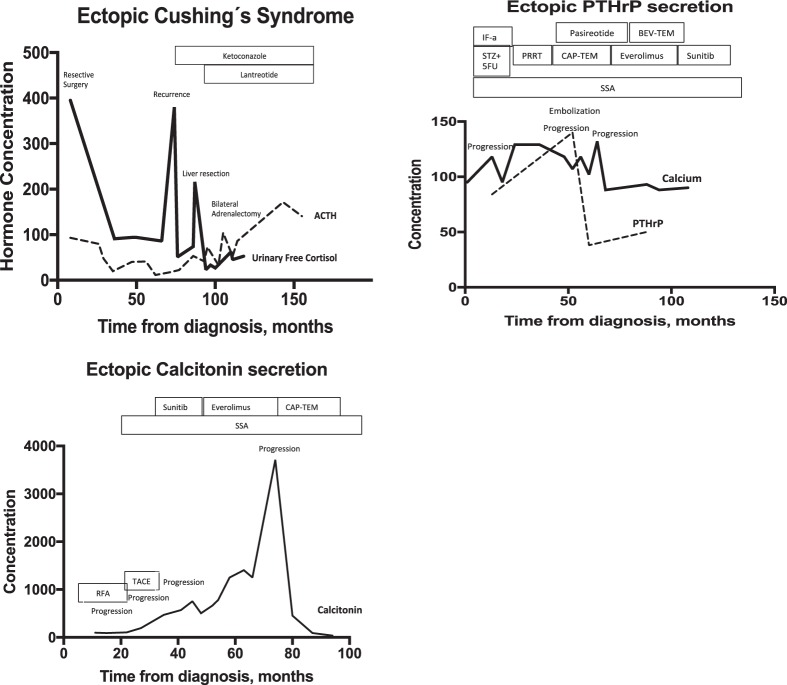
*XY* plots on different ePNS with patient examples. **a** ECS hormonal status [ACTH (pg/ml), urinary-free cortisol (UFC, pg/g creatinine)]) over time. **b** Ectopic PTHrP secretion and plasma calcium concentration [PTHrP (pg/ml), plasma calcium (mg/dl)]) over time. **c** VIP hormonal status (pmol/l) over time. **d** Calcitonin (pg/ml) hormonal status over time

All 12 patients with up to Stage III disease underwent surgery with the intention to cure and/or control the ectopic hormonal excess. Two patients with distant stage disease underwent debulking surgery to control EPNS (Supplementary Table 3). Eight patients with ECS, all of whom with NENs of thoracic origin, underwent bilateral adrenalectomy in order to control hypercortisolaemia early in the disease course. Anti-proliferative treatments including somatostatin analogues (SSA), systemic chemotherapy and targeted therapies were used as indicated during the disease course (Supplementary Table 3). Additionally, symptomatic treatments, (e.g. intravenous (IV) fluids, bisphosphonates (IV) and cinacalcet in the case of hypercalcaemia due to PTHrP secretion, and ketoconazole/metyrapone in six cases with ECS), were given as indicated. Generally, variable effects for different lines of treatment in terms of controlling hormonal excess were achieved (Fig. 4). In the majority of patients, hormone over-secretion was related to disease recurrence or progressive disease.

## Discussion

In this study, the prevalence of the EPNS secondary to a variety of NENs as obtained from the Greek NEN database (EKPA-Laiko) was found to be 1.9% and included five different syndromes. In contrast, EPNS was more prevalent in patients with LCs (6.7%) in both centres and was mainly related to ECS. PanNENs exhibited the largest diversity, as they were involved in three EPNS with a prevalence of 1.9%. All patients with EPNS secondary to NENs were related to well-differentiated tumours, with the majority presenting with Grade 2 disease. Atypical histopathological findings were prevalent among patients with ECS secondary to LC (7/12). In contrast to ECS, where the majority of patients had locoregional disease at diagnosis mostly attributed to lung NENs, all patients with hypercalcitonaemia, PTHrP and/or β-HCG secretion had Stage IV disease.

The diagnosis of EPNS was synchronous in six patients, metachronous in five patients and preceded the NEN diagnosis in the remaining 10 patients, with the latter only related to ECS, mainly of LC origin (9/10). Elevated ectopic hormone secretion was related to disease recurrence or progression in the majority of patients. The response to treatment in relation to secretory syndromes varied greatly among the patients included in the study. In the single patient with hypercalcaemia due to PTHrP secretion, surgery, multiple anti-proliferative treatments and symptomatic therapy with IV fluids, biphosphonate and cinacalcet controlled the secretory symptoms adequately. Resective surgery, when feasible, combined with anti-proliferative/symptomatic treatments as indicated per patient, and occasional bilateral adrenalectomy, achieved control of the hypercortisolaemia.

Interestingly, patients presenting with EPNS prior to their NEN diagnosis had longer EFS compared to patients with synchronous or metachronous EPNS. Importantly, ECS of extra-thoracic origin was more often synchronous or metachronous, and exhibited worse prognosis compared to patients with ECS of lung or thymic origin. In particular, the diagnosis of ECS was overt or covert in many cases (10/16), facilitating diagnosis of NEN at an earlier stage, mostly regarding LC-related ECS. Indeed, a minority of four patients (4/16) with ECS had Stage IV disease at diagnosis, only one of whom had LC-related ECS.

Generally, mortality rates might be increased in patients with ECS and uncontrollable hypercortisolaemia, partly due to susceptibility to infection and thromboembolic episodes [15, 25–28]. This increased mortality risk was not confirmed in patients with LC-related ECS of our study, as comparable OS and EFS rates were evident between LC patients with and without ECS. This was possibly due to overrepresentation of LC patients with early-stage disease (I–III) and mild-related hypercortisolism, who underwent curative surgery early in the disease course. On the contrary, dismal outcomes were clearly evident in the few cases of ECS of extra-thoracic origin that was presented at stage IV with synchronous or metachronous ECS.

Although elevated calcitonin is a marker of medullary thyroid carcinoma (MTC), it may be ectopically produced and can be elevated in other NENs, particularly those originating from the foregut. The immunohistochemical and morphologic profile of calcitonin-secreting NENs may be indistinguishable from MTC [14]. Presenting symptoms related to hypercalcitoninaemia may include profound diarrhoea, metabolic alkalosis with hypokalaemia, hypocalcaemia, hypomagnesaemia, hypophosphataemia and hyperglycaemia [29]. In the majority of cases in the literature, hypercalcitonaemia is not associated with a secretory, clinically apparent syndrome. In our study, all four cases detected with hypercalcitonaemia were described together with other EPNS diagnoses. These patients manifested diarrhoea and flushing at some point and had no other measurable bioactive hormonal secretion. However, the main mechanism of diarrhoea in these patients maybe a shortened colonic transit time resulting in decreased absorption, rather than a direct calcitonin-induced small intestinal fluid secretion [30]. Importantly, all the patients with ectopic secretion of calcitonin had disseminated disease at diagnosis.

Ectopic hypersecretion of PTHrP by NENs is indeed rare, as demonstrated in our study, and seems to be associated exclusively with metastatic panNENs in accordance with previous reports [13]. PTHrP production may also have a major clinical impact due to poorly controllable hypercalcaemia with increased associated morbidity and mortality. The single case with ectopic PTHrP secretion included in our study required aggressive multimodal treatment in order to control the hypercalcaemia.

Ectopic β-HCG secondary to thoracic tumours (mainly SCLCs, carcinoids and extragonadal germ cell tumours), clear cell renal tumours or liver carcinomas, may, on rare occasions, induce gynaecomastia in men, menstrual irregularity and virilisation in women, and precocious puberty in children [19]. In our study, one male patient with a primary of unknown origin demonstrated dual secretion of calcitonin and β-HCG that was not associated with overt clinical symptoms. Thus, β-HCG was regardered as biologically active, as gonadotrophin levels were suppressed in the presence of high testosterone levels and recovered following successful treatment of the neoplasm and subsequent normalisation of β-HCG levels. Additionally, IHC staining of metastatic lesions in the liver was positive for β-HCG, calcitonin, synaptophysin and Chromogranin A.

Importantly, apart from NENs of unknown primary origin, EPNS were mainly present in NENs of the foregut, i.e. panNENs, LCs and thymic carcinoids, with the exception of one patient with ECS secondary to a small intestinal neuroendocrine tumour. PanNENs in particular were involved in a diverse spectrum of three different EPNS and were often disseminated at diagnosis. This is in accordance with the diversity of panNEN hormone secretion at a late stage and the extreme scarcity of EPNS secondary to midgut tumours, in particular small intestinal NENs as reported by Crona et al. in a study on multiple and secondary hormone secretion from gastroentero-pancreatic NENs [31].

In the majority of patients, hormone oversecretion was related to disease recurrence or progressive disease. Thus, plasticity of the associated ectopically produced hormones during follow-up may be useful as prognostic and predictive markers. In ECS, ACTH and cortisol levels were also affected by anti-hormonal treatments and bilateral adrenalectomy. Hence, response to anti-proliferative treatments was rather poorly depicted in cortisol and ACTH levels (Fig. 4A). As the prevalence of EPNS is relatively low, it mainly concerns NENs of the foregut; and with regards to LC-related ECS no clear association to unfavourable prognosis was evident in our series, we cannot advocate in favour of routine screening for EPNS in NEN patients. Nevertheless, a multidisciplinary approach within a specialised referral centre for NENs is crucial and should be considered standard of care for all NEN patients, as it may even facilitate early EPNS detection and implementation of multimodal individualised treatment plans in such complex cases.

Our study has some limitations. The scarcity of EPNS secondary to NENs combined with the long OS of NEN patients makes any effort to undertake a prospective randomised study on patient outcomes virtually impossible. Indeed, due to the small sample size of this study, survival analysis findings should be interpreted with caution, precluding safe conclusions to be derived. NEN heterogeneity, treatment factors, such as the quality of surgery in different centres, differences in systemic therapies as well as multiple treatments, may all have confounded the results. Ectopic hormonal screening was not performed on a regular basis, but as indicated per patient according to the existing guidelines. Thus, the results may differ from prospectively collected data, and the prevalence of EPNS causing s subclinical symptomatology may have been underestimated. This study is also limited by a referral bias to the two tertiary centres involved. Some of these drawbacks are, of course, inherent to any retrospective multicentre study. However, the strength of this study is that it provides a comprehensive epidemiologic picture of NEN associated with rare EPNS with a prevalence assessment and a special focus on LC-related ECS. Additionaly, it demonstrates that timing of EPNs diagnosis and origin of the primary tumoural source may impact NEN prognosis.

Systematic documentation and registration of these syndromes in national databases and international collaborations between referral centres may increase awareness and further elucidate several aspects of EPNS. In particular, recent diagnostic and therapeutic advances in the field of NENs, might help to clearly delineate EPNS in NENs, shed light to EPNS pathogenesis, facilitate earlier tumour/EPNS diagnosis, improve patient outcomes by individualised therapeutic choices and possibly avoid unnecessary screening and associated costs by implementing surveillance approaches as clinically indicated per patient.

## Electronic supplementary material

Supplementary Table1: Patients with different NEN types included in the study.

Supplementary Table2: Criteria for defining Paraneoplastic Endocrine Syndrome (EPNS).

Supplementary Table3: Patient treatments at diagnosis and during follow-up.

Supplementary Figure 1A: Time elapsed from NEN diagnosis to ectopic hormone secretion in patients with synchronous or metachronous EPNS; and Supplementary Figure 1B: Time elapsed from EPNS diagnosis to NEN diagnosis in patients presenting with EPNS first (cases with overt or covert Ectopic Cushing's Syndrome).
