# Supplementary material for: Endocrine paraneoplastic syndromes in patients with neuroendocrine neoplasms
Source: Endocrine. 2018 Oct 2;64(2):384–92. doi: 10.1007/s12020-018-1773-3 (PMC6531606; doi:10.1007/s12020-018-1773-3)
Supplement: Supplementary file 1 — Supplementary Table1: Patients with different NEN types included in the study. [file 12020_2018_1773_MOESM1_ESM.docx]

Supplementary Table 1. Patients with different NEN types included in the study

| NEN type | N (total=834) |
| --- | --- |
| Gastric | 141 |
| Duodenal | 36 |
| Pancreatic | 265 |
| Small Intestine | 79 |
| Appendix | 49 |
| Colon | 8 |
| Rectal | 33 |
| Lung | 164 |
| Thymic | 2 |
| Uknown Primary Origin | 41 |
| Various other origin (ovarian, prostate, larynx) | 16 |
