# Supplementary material for: Endocrine paraneoplastic syndromes in patients with neuroendocrine neoplasms
Source: Endocrine. 2018 Oct 2;64(2):384–92. doi: 10.1007/s12020-018-1773-3 (PMC6531606; doi:10.1007/s12020-018-1773-3)
Supplement: Supplementary file 2 — Supplementary Table2: Criteria for defining Paraneoplastic Endocrine Syndrome (EPNS). [file 12020_2018_1773_MOESM2_ESM.docx]

*Supplementary Table 2.* Criteria for defining Paraneoplastic Endocrine Syndrome (ePNS).

| 1. Endocrine or metabolic disturbance in a patient with a NEN |
| --- |
| 2. Remission after successful treatment |
| 3. Return of endocrine syndrome with tumor recurrence |
| 4. Abnormally regulated elevated hormone levels |
| 5. Significant gradient between hormone concentration in the venous effluent from the tumor and arterial hormone levels |
| 6. Extracts from tumor exhibit bio- and/or immunoreactive hormone |
| 7. Relevant hormone mRNA can be identified in tumor tissue |
| 8. Synthesis and secretion of relevant hormone by tumor cells in vitro |
