# Supplementary material for: Endocrine paraneoplastic syndromes in patients with neuroendocrine neoplasms
Source: Endocrine. 2018 Oct 2;64(2):384–92. doi: 10.1007/s12020-018-1773-3 (PMC6531606; doi:10.1007/s12020-018-1773-3)
Supplement: Supplementary file 3 — Supplementary Table3: Patient treatments at diagnosis and during follow-up. [file 12020_2018_1773_MOESM3_ESM.docx]

*Supplementary Table 3.* Patient treatments at diagnosis and during follow-up.

| **Resective Surgery of the Primary Tumor** |  |
| --- | --- |
| Yes | 13 |
| No | 8 |
| **Systemic anti-cancer treatments** |  |
| Chemotherapy | 7 |
| Targeted agents (TKI and/or mTOR inhibitors) | 3 |
| Somatostatin Analogues | 5 |
