# Supplementary figures and images for: Endocrine paraneoplastic syndromes in patients with neuroendocrine neoplasms
Source: Endocrine. 2018 Oct 2;64(2):384–92. doi: 10.1007/s12020-018-1773-3 (PMC6531606; doi:10.1007/s12020-018-1773-3)

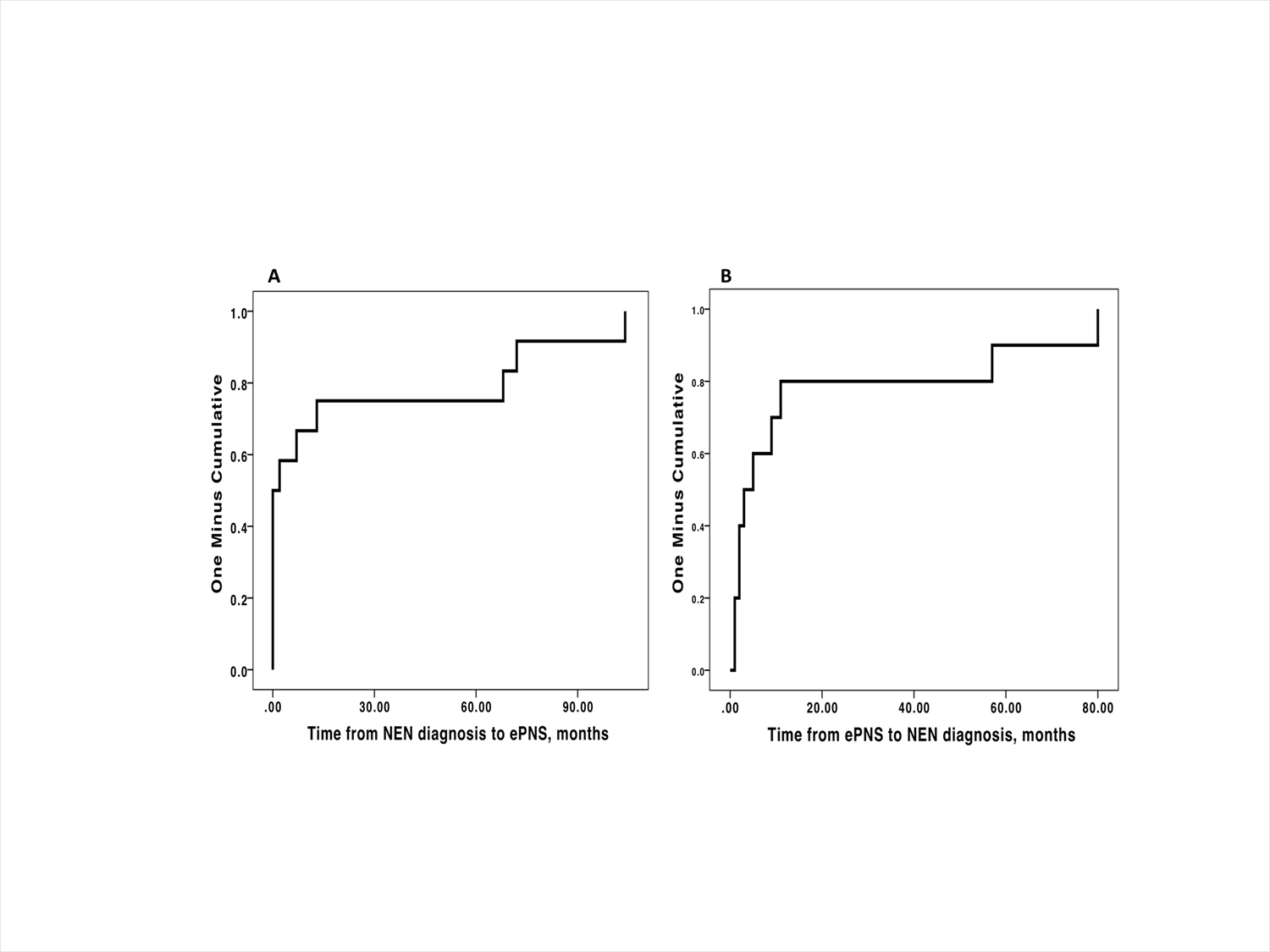

Supplement: Supplementary file 4 — Supplementary Figure 1A: Time elapsed from NEN diagnosis to ectopic hormone secretion in patients with synchronous or metachronous EPNS; and Supplementary Figure 1B: Time elapsed from EPNS diagnosis to NEN diagnosis in patients presenting with EPNS first (cases with overt or covert Ectopic Cushing's Syndrome). [file 12020_2018_1773_MOESM4_ESM.png]
